# A review of the spider-attacking *Polysphinctadizardi* species-group (Hymenoptera, Ichneumonidae, Pimplinae), with descriptions of seven new species from South America

**DOI:** 10.3897/zookeys.1041.65407

**Published:** 2021-06-01

**Authors:** Diego G. Pádua, Ilari E. Sääksjärvi, Tamara Spasojevic, Kari M. Kaunisto, Ricardo F. Monteiro, Marcio L. Oliveira

**Affiliations:** 1 Programa de Pós-Graduação em Entomologia, Instituto Nacional de Pesquisas da Amazônia (INPA), Manaus, Brazil Instituto Nacional de Pesquisas da Amazônia Manaus Brazil; 2 Biodiversity Unit, Zoological Museum, University of Turku, Turku, Finland University of Turku Turku Finland; 3 Department of Entomology, National Museum of Natural History, Washington, DC, USA National Museum of Natural History Washington United States of America; 4 Laboratório de Ecologia de Insetos, Depto. de Ecologia, Universidade Federal do Rio de Janeiro (UFRJ), Rio de Janeiro, Brazil Universidade Federal do Rio de Janeiro Rio de Janeiro Brazil

**Keywords:** Amazonia, Andes, Brazil, Darwin wasps, ectoparasitoid, Ecuador, Ephialtini, koinobiont, Neotropics, Peru, *Polysphincta* genus group, parasitoid, rainforest

## Abstract

The Neotropical *Polysphinctadizardi* species-group is revised. We describe seven new species from South America: *P.bonita***sp. nov.**, *P.cosnipata***sp. nov.**, *P.inca***sp. nov.**, *P.macroepomia***sp. nov.**, *P.organensis***sp. nov.**, *P.pichincha***sp. nov.**, and *P.teresa***sp. nov.** In addition, we provide a diagnosis and an identification key to all species of the group.

## Introduction

*Polysphincta* Gravenhorst, 1829 is a Neotropical and Holarctic Darwin wasp genus with 30 valid species (Yu et al. 2016; Kloss et al. 2018; Higa and Penteado-Dias 2020). The genus belongs to the *Polysphincta* genus-group (*sensu*Gauld and Dubois 2006) which exclusively comprises koinobiont ectoparasitoids of spiders (Matsumoto 2016; Yu et al. 2016; Kloss et al. 2018).

The revision of the Neotropical species of *Polysphincta* was started by Gauld (1991) and Gauld et al. (1998) who described several new species from Central America and divided the genus into three species-groups based on morphological characters: *P.dizardi*, *P.gutfreundi*, and *P.purceli* species-groups. Gauld (1991) also reported that several undescribed species occur throughout tropical America.

The species of the *P.dizardi* species-group are somewhat intermediate between the “more typical” *Polysphincta* species and the species of *Hymenoepimecis* Viereck (Gauld 1991). The *P.dizardi* species-group is normally characterized by the following two characters: a shelf-like projection (pronotal shelf) in the mediodorsal part of the pronotum and epomia absent.

The morphological phylogenetic analysis of the *Polysphincta* genus-group by Gauld and Dubois (2006) placed a single included representative of *P.dizardi* species-group, *P.shabui* Gauld, into the clade “F” as a sister group of genera *Ticapimpla* Gauld, *Acrotaphus* Townes and *Hymenoepimecis*. This suggests that the *P.dizardi* species-group could be a new genus. The status of *Polysphincta* should be revised after the tropical fauna of the genus is better known.

During the last two decades, we have found several new species of *Polysphincta* from various parts of South America (tropical Andes, Amazonia, Brazilian coastal rain forests and Chilean temperate rain forests), which calls for the revision of Neotropical species of the genus. The review of the *P.dizardi* species-group, studied here, is the first part of this larger work.

## Materials and methods

The specimens studied in this review are deposited in the following collections:

**BMNH**The Natural History Museum, London, United Kingdom;

**DCBU**Departamento de Ecologia e Biologia Evolutiva, São Carlos, São Paulo, Brazil;

**INPA**Invertebrate Collection of the Instituto Nacional de Pesquisas da Amazônia, Manaus, Amazonas, Brazil;

**MUSM** Universidad Nacional de San Marcos, Lima, Peru;

**MZUSP**Zoological Museum of the Universidade de São Paulo, São Paulo, São Paulo, Brazil;

**RBINS**Royal Belgian Institute of Natural Sciences, Brussels, Belgium;

**UEFS**Universidade Estadual de Feira de Santana, Feira de Santana, Bahia, Brazil;

**UFMG**Universidade Federal de Minas Gerais, Belo Horizonte, Minas Gerais, Brazil;

**ZMUT**Biodiversity Unit, Zoological Museum of the University of Turku, Turku, Finland.

The morphological terminology follows Broad et al. (2018) and style of the descriptions follow those of Gauld (1991). However, we add two new characters to the descriptions: the shape of the tarsal claws and the shape of the pronotal shelf. We also add the following proportions to the descriptions: (a) margin of gena/length of eye; (b) length of the epomia/length of the proximal mandibular width; and (c) length/posterior width of tergite II.

The measures and proportions between the structures are given as the value of the holotypes or paratypes [in brackets], followed by the minimum and maximum number of variations. The [brackets] were also used to add, supplement or correct information on the specimen labels.

Specimens were examined using OLYMPUS SZ61 and SZX10 (at ZMUT) and the ZEISS Stemi 2000 (at INPA) stereomicroscopes. Measurements were obtained using millimetric oculars attached to the stereomicroscope, calibrated with a precision ruler. Digital images were taken using a CANON DS126461 digital camera attached to an OLYMPUS SZX16 stereomicroscope and combined by using the software Zerene Stacker (v. 1.04 Build T201706041920) (at ZMUT) and a LEICA DMC4500 digital camera attached to a LEICA M205A stereomicroscope and combined by using the software Helicon Focus v. 5.3 Pro. (at INPA).

The distributional maps were created using SimpleMappr online software (Shorthouse 2010).

## Taxonomy

### The *Polysphinctadizardi* species-group

**Diagnosis.** The *P.dizardi* species-group can be distinguished from all other species-groups of the genus by the combination of two characters: (1) pronotum with a strong shelf-like projection mediodorsally and (2) submetapleural carina absent.

**Remarks.** According to Gauld (1991) and our new discoveries, this species group is known to occur only in the Neotropical region.

#### Key to the species of the *P.dizardi* species-group

[Obs. Only the males of *P.shabui* Gauld, *P.sinearanea* Pádua, and *P.organensis* sp. nov. are known].

**Table:** 

1	Epomia present (Figs 5B, 9B, 10B)	**2**
–	Epomia absent (Figs 1B, 2B, 3B, 4B, 6B, 7B, 8B)	**4**
2	Epomia 1.5 times the length of the proximal mandibular width (Fig. 5B)	***P.macroepomia* sp. nov.**
–	Epomia <1.0 times the length of the proximal mandibular width (Figs 9B, 10B)	**3**
3	Metasoma orange, with posterior margins of tergites II–IV narrowly black, posterior half of tergite V black, and tergites VI+ black (Fig. 9A, C); ovipositor robust (Fig. 9A)	***P.sinearanea* Pádua, 2018**
–	Metasoma darkish brown, with posterior margins of tergites II–V narrowly black (Fig. 10A, C); ovipositor slender (Fig. 10A)	***P.teresa* sp. nov.**
4	Metasoma orange with posterior margins of tergites II–IV narrowly black, tergites V+ or VI+ black (Figs 4A, 7A); fore wing yellowish hyaline with or without apex slightly blackish (Figs 4A, 7A); mesosoma entirely orange (Figs 4A, 7A)	**5**
–	Metasoma entirely darkish brown (some specimens with tergites I–III reddish orange with posterior margin blackish) or blackish with anterior parts whitish (Figs 1A, 2A, 3A, 6A, 8A); fore wing hyaline (Figs 1A, 2A, 3A, 6A, 8A); mesosoma entirely orange or reddish brown, or orange or reddish brown with black parts (Figs 1A, 2A, 3A, 6A, 8A)	**6**
5	Malar space >0.6 times as long as proximal mandibular width; hind coxa black (Fig. 4A)	***P.inca* sp. nov.**
–	Malar space 0.4 times as long as proximal mandibular width; hind coxa orange (Fig. 7A)	***P.pichincha* sp. nov.**
6	Mesosoma reddish brown or orange with some blackish or brownish markings (Figs 2A, 3A)	**7**
–	Mesosoma entirely reddish brown or orange without blackish or brownish markings (Figs 6A, 8A)	**8**
7	Mesosoma reddish brown with anterior part of pronotum, propleuron, metapleuron and propodeum blackish (Fig. 3A); metasoma entirely darkish brown (Fig. 3A, C)	***P.dizardi* Gauld, 1991**
–	Mesosoma orange with metapleuron and propodeum brown (Fig. 2A); metasoma brownish with anterior and anterolateral margins of tergites III–V whitish (Fig. 2A, C)	***P.cosnipata* sp. nov.**
8	Metasoma entirely darkish brown (Fig. 8A, C) or darkish brown with tergites I–III reddish orange with posterior margin blackish; malar space 0.6 times as long as proximal mandibular width	***P.shabui* Gauld, 1991**
–	Metasoma blackish with tergites II–IV or II–VI with anterior and anterolateral margins whitish (Figs 1A, 6A, C); malar space <0.5 times as long as proximal mandibular width	**9**
9	Metasoma blackish with tergites II–VI with anterior and anterolateral margins whitish (Fig. 6A, C); ovipositor 1.2–1.3 times as long as hind tibia	***P.organensis* sp. nov.**
–	Metasoma with tergite I orange with posterior margin black, tergites II–IV brownish with anterior and anterolateral margins whitish and posterior margin black, and tergites V+ brownish (Fig. 1A, E); ovipositor 1.0 times as long as hind tibia	***P.bonita* sp. nov.**

### The species of *Polysphinctadizardi* species-group

#### Polysphincta bonita

Taxon classificationAnimaliaHymenopteraIchneumonidae

Pádua & Sääksjärvi sp. nov.

15C2F130-C914-5E09-8DB4-B8C203FB71EF

http://zoobank.org/29EA486A-7AD0-4C27-BB9A-F54C4B55E11A

Fig. 1A–E

##### Diagnosis.

**Figure 1.**
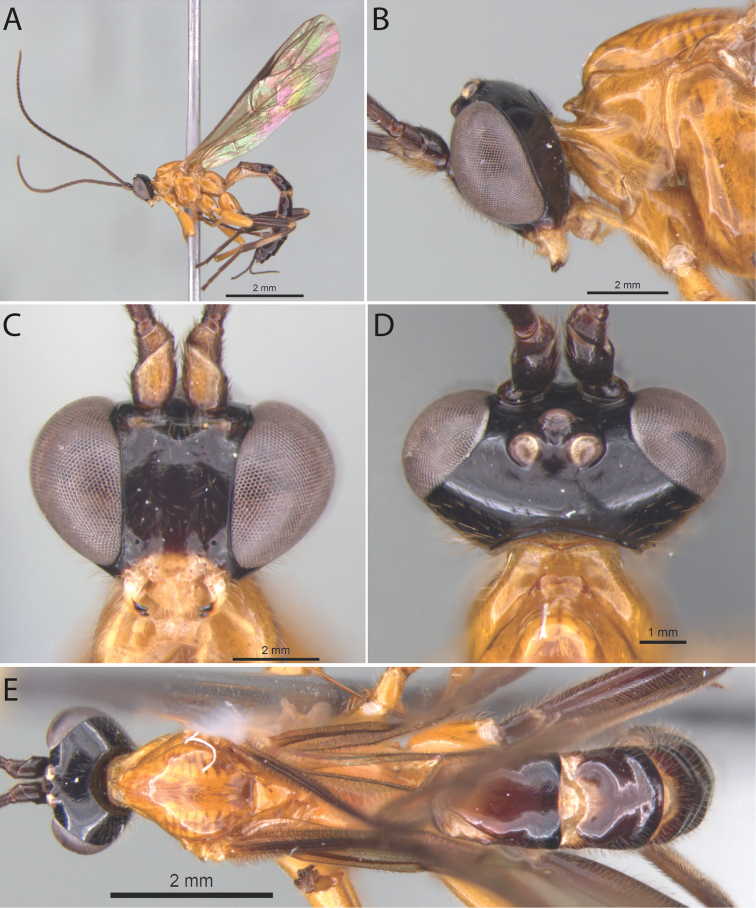
*Polysphinctabonita* sp. nov., ♀, holotype **A** habitus, lateral view **B** head and pronotum, lateral view **C** face, anterior view **D** head and shelf-like projection, dorsal view **E** habitus, dorsal view.

**Figure 2.**
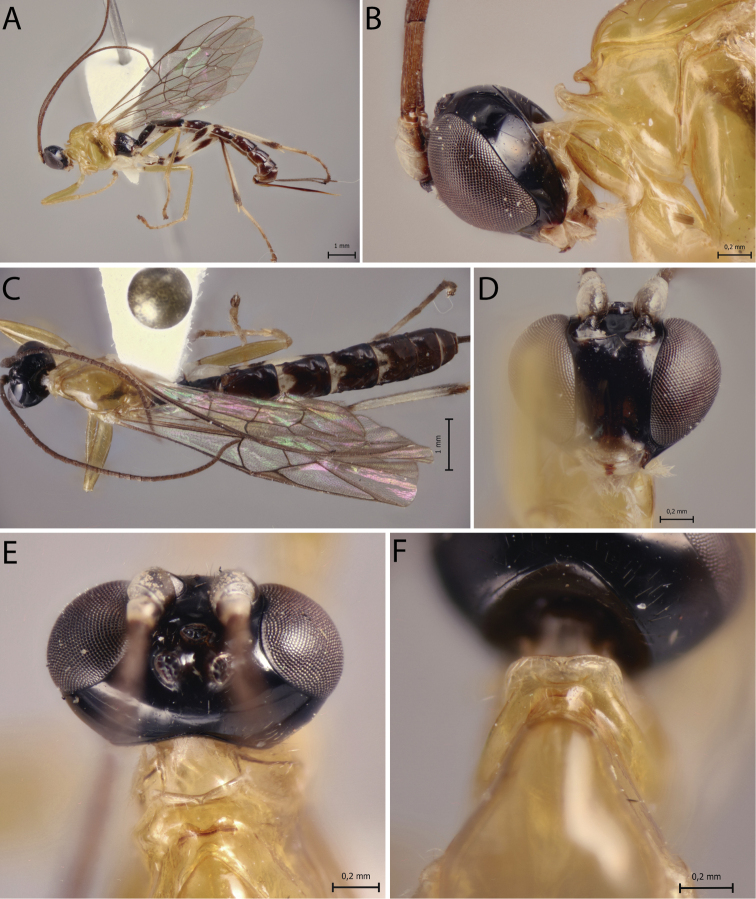
*Polysphinctacosnipata* sp. nov., ♀, holotype **A** habitus, lateral view **B** head and pronotum, lateral view **C** habitus, dorsal view **D** face, anterior view **E** head, dorsal view **F** shelf-like projection, dorsal view.

**Figure 3.**
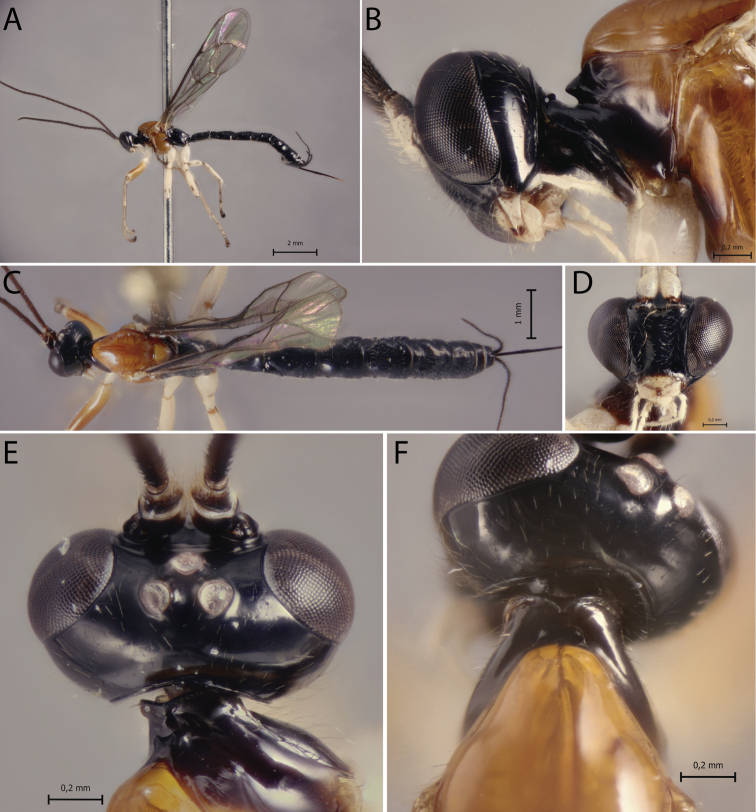
*Polysphinctadizardi* Gauld, 1991, ♀, paratype **A** habitus, lateral view **B** head and pronotum, lateral view **C** habitus, dorsal view **D** face, anterior view **E** head, dorsal view **F** shelf-like projection, dorsal view.

*Polysphinctabonita* sp. nov. can be distinguished from other species of the *P.dizardi* species-group by the combination of the following characters: (1) epomia absent (Fig. 1B); (2) malar space 0.5 times as long as proximal mandibular width (Fig. 1B); (3) fore wing vein 1*cu-a* interstitial relative to *M&RS*; (4) mesosoma orange (Fig. 1A); (5) wing hyaline, slightly infuscate (Fig. 1A); (6) hind leg brownish, except coxa orange and middle inner and outer region whitish (Fig. 1A); (7) metasoma with tergite I orange with posterior margin black, tergites II–IV brownish with anterior and anterolateral margins whitish and posterior margin black, and tergites V+ brownish (Fig. 1A, E); (8) ovipositor slightly slender, 1.0 times as long as hind tibia.

##### Description.

**Female.** Body about [9.5] mm. ***Head*.** Clypeus weakly convex, the posterior margin thin and straight centrally; malar space [0.5] times as long as proximal mandibular width; lower face [1.1] times as broad as high, weakly convex centrally, polished, with fine sparse setiferous punctures; head in dorsal view with margin of the gena weakly convex behind the eyes, and its margin about [0.5] times length of eye in dorsal view; ocelli moderately large, the lateral one separated from compound eyes by [1.1] times their own maximum diameter. ***Mesosoma*.** Pronotum without epomia; shelf-like projection, in dorsal view, with the apex bilobed, and in lateral view, with anterolateral part posteriorly rounded and weakly decurved; mesoscutum more or less robust, in dorsal view, smooth and polished, with notauli weakly impressed anteriorly; scutellum convex, not laterally carinate; mesopleuron highly polished, virtually impunctate; epicnemial carina reaching almost to the level of the lower corner of pronotum; epicnemium with a vestigial vertical carina near lower corner of pronotum; metapleuron convex, smooth and polished, with sparse, fine bristles evenly spaced, without a discernible submetapleural carina. Propodeum mediodorsally smooth and polished, with longitudinal carinae present only posteriorly and with scattered fine bristles. Fore wing length about [8.0] mm; 1*cu-a* interstitial relative to *M&RS*; base of 1*m-cu&M* separated from *CU* by about length of 2*cu-a*; hind wing with distal abscissa of *CU* present and complete, well pigmented; first abscissa of *RS* subequal to *rs-m*. Tarsal claw with proximal lobe quadrangular, with claw apex slightly overtaking the distal margin of lobe. ***Metasoma*.** Tergite I [1.25] times as long as posteriorly broad, dorsally with lateromedian longitudinal carinae only discernible at the extreme anterior part; sternite I with a weak swelling near the hind rim, and with a weak median longitudinal ridge anteriorly; tergite II about [1.25] times as long as posteriorly broad, highly polished, at most with only fine setiferous punctures laterally; tergite III about [1.1] times as long as posteriorly broad, highly polished, at most with only fine setiferous punctures; subgenital plate subquadrate. Ovipositor slightly slender, about [1.0] times as long as hind tibia, posteriorly evenly tapered to a sharp point.

##### Color.

Head black except posterior 0.8 of clypeus yellowish; antennae brownish with scape and pedicel ventrally yellowish; mouthparts whitish, except apex of mandible blackish. Mesosoma orange. Metasoma with tergite I orange with posterior margin black, tergites II–IV brownish with anterior and anterolateral margins whitish and posterior margin black, and tergites V+ brownish. Fore and mid leg orange, hind leg brownish, except coxa orange and a medium inner and outer region whitish. Wings are hyaline, slightly infuscate, pterostigma brown. Ovipositor brown, with posterior and anterior part whitish.

**Male.** Unknown.

##### Type material.

***Holotype*** ♀. Brazil, BA [= Bahia], Camacan, PPPN [sic] [= RPPN, Reserva Particular do Patrimônio Natural], Serra Bonita, IX.2010, Malaise trap 3 (without collector), UEFS.

##### Distribution.

Brazil (Fig. 13).

##### Biological note.

Host unknown.

##### Etymology.

The specific name (in apposition) refers to the type locality of this species, RPPN Serra Bonita, Bahia state, Brazil, and also to the beauty of this new species.

##### Remarks.

*Polysphinctabonita* sp. nov. closely resembles *P.organensis* sp. nov. mainly by the coloration, with mesosoma entirely orange and metasoma brownish with tergites II–IV or II–VI whitish in anterior and anterolateral margins. It clearly differs from *P.organensis* sp. nov. by having ovipositor 1.0 times as long as hind tibia and fore and mid leg orange, hind leg brownish, except coxa orange and a medium inner and outer region whitish (ovipositor >1.2 times as long as hind tibia and fore leg orange, mid leg orange with coxa, trochanter and trochantellus whitish and tarsus brownish, hind leg whitish with coxa inner region, trochanter proximally, trochantellus distal, femur proximally and distally, tibia proximally and distally, first tarsal segment distally, and remaining tarsal segments entirely blackish brown in *P.organensis* sp. nov.).

#### Polysphincta cosnipata

Taxon classificationAnimaliaHymenopteraIchneumonidae

Pádua & Sääksjärvi sp. nov.

D157D343-1E17-564E-B610-8C1C6116226F

http://zoobank.org/1D4251B0-17C6-4220-AF11-9B2D2E7FB721

Fig. 2A–F

##### Diagnosis.

**Figure 4.**
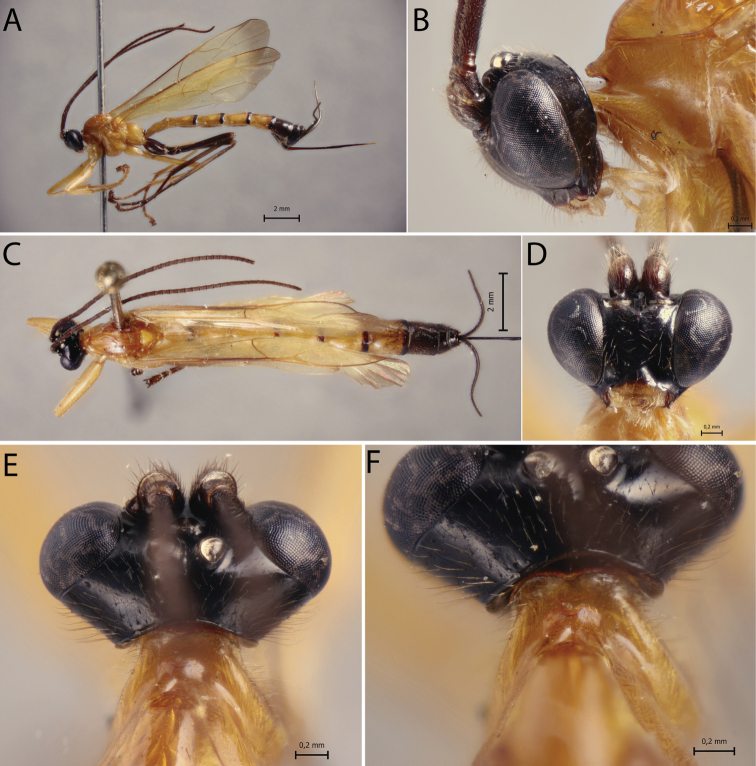
*Polysphinctainca* sp. nov., ♀, holotype **A** habitus, lateral view **B** head and pronotum, lateral view **C** habitus, dorsal view **D** face, anterior view **E** head, dorsal view **F** shelf-like projection, dorsal view.

**Figure 5.**
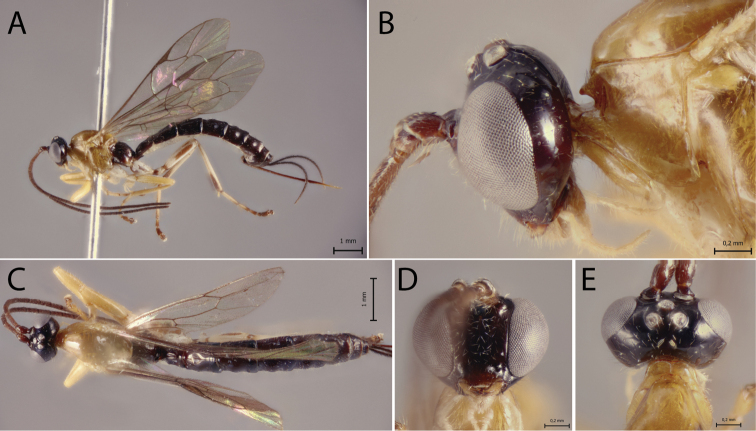
*Polysphinctamacroepomia* sp. nov., ♀, holotype **A** habitus, lateral view **B** head and pronotum, lateral view **C** habitus, dorsal view **D** face, anterior view **E** head and shelf-like projection, dorsal view.

**Figure 6.**
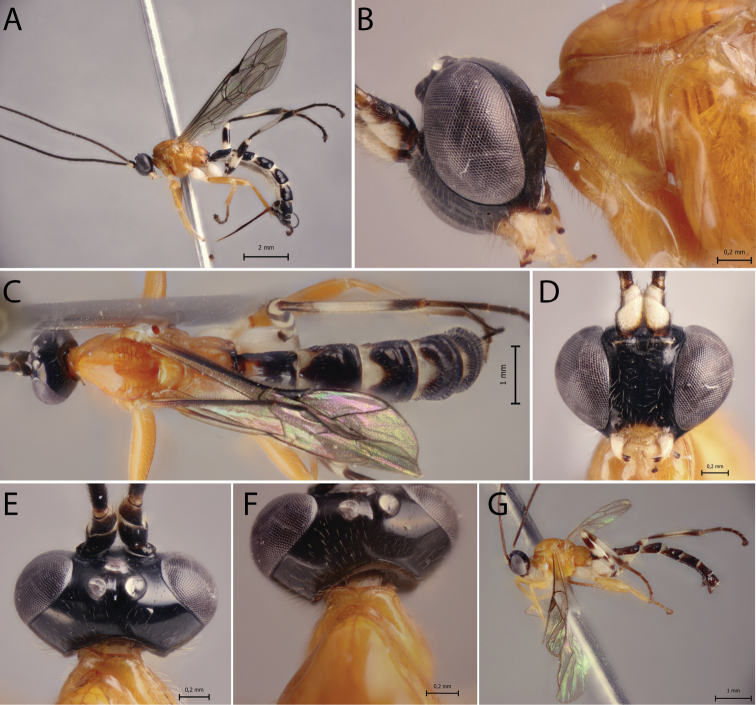
*Polysphinctaorganensis* sp. nov. **A** habitus, lateral view, ♀ (holotype) **B** head and pronotum, lateral view, ♀ (holotype) **C** habitus, dorsal view, ♀ (holotype) **D** face, anterior view, ♀ (holotype) **E** head, dorsal view, ♀ (holotype) **F** shelf-like projection, dorsal view, ♀ (holotype) **G** habitus, ♂ (paratype).

**Figure 7.**
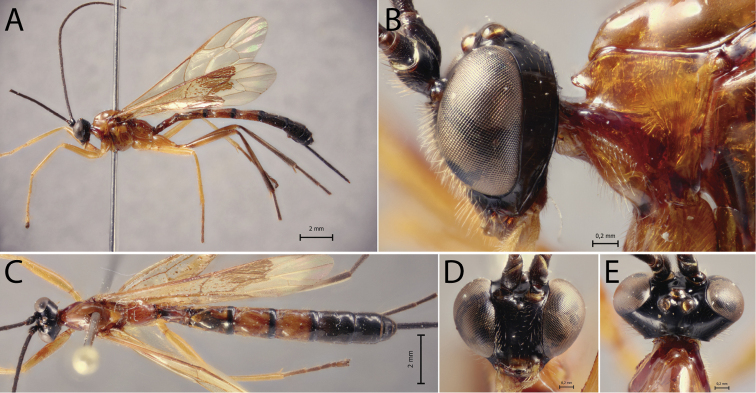
*Polysphinctapichincha* sp. nov., ♀, holotype **A** habitus, lateral view **B** head and pronotum, lateral view **C** habitus, dorsal view **D** face, anterior view **E** head and shelf-like projection, dorsal view.

**Figure 8.**
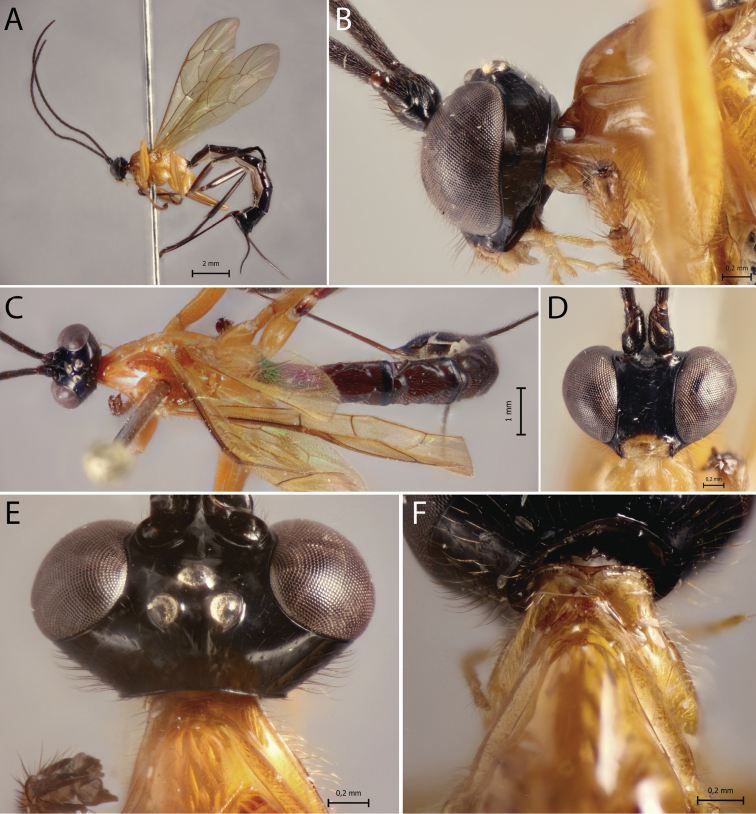
*Polysphinctashabui* Gauld, 1991, ♀, paratype **A** habitus, lateral view **B** head and pronotum, lateral view **C** habitus, dorsal view **D** face, anterior view **E** head, dorsal view **F** shelf-like projection, dorsal view.

*Polysphinctacosnipata* sp. nov. can be distinguished from other species of the *P.dizardi* species-group by the combination of the following characters: (1) epomia absent (Fig. 2B); (2) malar space 0.4 times as long as proximal mandibular width (Fig. 2B); (3) fore wing vein 1*cu-a* more or less interstitial relative to *M&RS* (Fig. 2A); (4) mesosoma orange with metapleuron and propodeum brown (Fig. 2A); (5) wings hyaline (Fig. 2A); (6) hind leg whitish with inner part of coxa, trochanter proximally, trochantellus distally, femur proximally and distally, tibia proximally and distally, first tarsal segment distally, and remaining tarsal segments entirely blackish brown (Fig. 2A); (7) metasoma brownish with anterior and anterolateral margins of tergites III–V whitish (Fig. 2A, C); (8) ovipositor slightly slender, 1.2 times as long as hind tibia.

##### Description.

**Female.** Body [8.0] mm. ***Head*.** Clypeus weakly convex, posterior margin thin and flat centrally; malar space [0.4] times as long as proximal mandibular width; lower face [0.9] times as broad as high, weakly convex centrally, polished, with fine sparse setiferous punctures; head in dorsal view with margin of the gena weakly convex behind eyes and its margin about [0.5] times length of eye; ocelli moderately large, lateral ones separated from compound eyes by about [0.8] times their own maximum diameter. ***Mesosoma*.** Pronotum without epomia; shelf-like projection, in dorsal view, bilobed, subquadrangular, and in lateral view, slender, with anterolateral corners weakly decurved; mesoscutum more or less robust, in dorsal view, smooth and polished, with notauli weakly impressed anteriorly; scutellum convex, not laterally carinate; mesopleuron highly polished, virtually impunctate; epicnemial carina reaching almost to level of lower corner of pronotum; epicnemium with vestigial vertical carina near lower corner of pronotum; metapleuron convex, smooth and polished, with sparse, fine bristles evenly spaced, without discernible submetapleural carina. Propodeum mediodorsally smooth and polished, with longitudinal carinae present only posteriorly and with scattered fine bristles. Fore wing length about [7.0] mm; 1*cu-a* more or less interstitial relative to *M&RS*; base of 1*m-cu&M* separated from *CU* by about length of 2*cu-a*; hind wing with distal abscissa of *CU* present and complete but weakly pigmented; first abscissa of *RS* subequal to *rs-m*. Tarsal claw with proximal lobe quadrangular, with claw apex slightly overtaking distal margin of lobe. ***Metasoma*.** Tergite I about [1.5] times as long as posteriorly broad, dorsally with lateromedian longitudinal carinae only discernible at extreme anterior part; sternite I with weak swelling near hind rim, and with weak median longitudinal ridge anteriorly; tergite II about [1.5] times as long as posteriorly broad, highly polished, at most with only fine setiferous punctures laterally; tergite III about [1.3] times as long as posteriorly broad, highly polished, at most with only fine setiferous punctures; subgenital plate subquadrate. Ovipositor slightly slender, [1.2] times as long as hind tibia, posteriorly evenly tapered to sharp point.

##### Color.

Head black except 0.8 of clypeus yellowish; antennae brownish with scape and pedicel ventrally whitish; mouthparts whitish, except apex of mandible brownish. Mesosoma orange with metapleuron and propodeum brown. Metasoma brownish with anterior and anterolateral margins of tergites III–V whitish. Fore leg orange, mid leg orange with coxa, trochanter and trochantellus whitish and tarsus distally brownish, hind leg whitish with coxa inner region, trochanter proximal, trochantellus distally, femur proximally and distally, tibia proximally and distally, first tarsal segment distally, and remaining tarsal segments entirely blackish brown. Wings hyaline, pterostigma brown. Ovipositor brown, with posterior and anterior parts whitish.

**Male.** Unknown.

##### Type material.

***Holotype*** ♀. Peru, CU [= Cusco], Cosñipata valley, San Pedro, 13°03'23"S, 71°32'55"W, 1520 m, 12.XII.2007, Malaise trap (C. Castillo leg.), MUSM.

##### Distribution.

Peru (Fig. 11).

##### Biological note.

Host unknown.

##### Etymology.

The specific name (in apposition) refers to type locality of this species, Cosñipata valley, Cusco, Peru.

##### Remarks.

*Polysphinctacosnipata* sp. nov. closely resembles *P.dizardi* Gauld, 1991 and *P.macroepomia* sp. nov. mainly by coloration, with mesosoma orange and propodeum blackish or brownish. However, it differs from *P.dizardi* by having pronotum orange and metasomal tergites II–VI with anterior and anterolateral margins whitish (anterior part of pronotum brownish and metasomal tergites entirely darkish brown in *P.dizardi*), and from *P.macroepomia* sp. nov. by having epomia absent (present in *P.macroepomia* sp. nov.).

#### Polysphincta dizardi

Taxon classificationAnimaliaHymenopteraIchneumonidae

Gauld, 1991

32F7C2F8-2DAD-5780-82D7-6765BAE5B17A

Fig. 3A–F

Polysphincta dizardi Gauld, 1991: 313. Holotype ♀, Costa Rica (MNCR).

##### Diagnosis.

*Polysphinctadizardi* can be distinguished from other species of the *P.dizardi* species-group by the combination of the following characters: (1) epomia absent (Fig. 3B); (2) malar space 0.45–0.5 times as long as proximal mandibular width (Fig. 3B); (3) fore wing vein 1*cu-a* interstitial relative to *M&RS* (Fig. 3A); (4) mesosoma reddish brown with anterior part of pronotum, propleuron, metapleuron and propodeum blackish (Fig. 3A); (5) wings hyaline (Fig. 3A); (6) hind leg whitish with femur laterally, tibia proximally and distally, and tarsus distally brownish (Fig. 3A); (7) metasoma entirely darkish brown (Fig. 3A, C); (8) ovipositor slender, 1.1–1.3 times as long as hind tibia.

##### Comments.

Additional characters to the original description (♀) are as follows: body about [7.7–8.3] 7.5–8.5; head in dorsal view with margin of the gena convex behind the eyes, and its margin [0.5–0.55] 0.45–0.55 times length of eye; shelf-like projection, in dorsal view, more or less developed anterolaterally, apex very weakly bilobed, and in lateral view, with anterolateral part of apex rounded and very weakly decurved; mesoscutum robust, in dorsal view; tarsal claw with proximal lobe quadrangular, with claw apex slightly overtaking distal margin of lobe.

##### Distribution.

Costa Rica (Fig. 11).

##### Biological notes.

Host unknown.

##### Materials examined.

***Paratypes*:** Costa Rica, Sn. José Pv., Zurqui de Moravis, 1600 m., nr. to Braulio Carrillo, I–II.1990 (Gauld leg.), 1♀, BMNH; idem, but Heredia Pv., 9.5 km., E. of El Tunel, 1000 m., IV.1989, 1♀, BMNH. **Costa Rica**: Sn. José Pv., Zurqui de Moravia, 1600 m. near to Braulio Carrillo, I.1991 (Gauld leg.), 1♀, BMNH; idem, but VI.1992, 1♀, BMNH.

#### Polysphincta inca

Taxon classificationAnimaliaHymenopteraIchneumonidae

Pádua, Sääksjärvi & Spasojevic sp. nov.

6B2387E5-26EC-518F-A7DC-B0A0DD0F6926

http://zoobank.org/8B54350E-46CE-4911-B8D6-4CC386F01AF6

Fig. 4A–F

##### Diagnosis.

*Polysphinctainca* sp. nov. can be distinguished from other species of the *P.dizardi* species-group by the combination of the following characters: (1) epomia absent (Fig. 4B); (2) malar space 0.6–0.7 times as long as proximal mandibular width (Fig. 4B); (3) fore wing vein 1*cu-a* interstitial relative to *M&RS* (Fig. 4A); (4) mesosoma orange, except posterior carinae of propodeum darkish brown (Fig. 4A); (5) wings yellowish hyaline with apex weakly blackish (Fig. 4A); (6) hind leg entirely darkish brown or darkish brown, with median region of tibia pale (Fig. 4A); (7) metasoma orange, with posterior margins (or only laterally) of tergites II–V narrowly black, tergites VI+ black (Fig. 4A, C); (8) ovipositor slender, 1.1–1.3 times as long as hind tibia.

##### Description.

**Female.** Body [13.0] 12.0–14.0 mm. ***Head*.** Clypeus weakly convex, posterior margin thin and flat centrally; malar space [0.7] 0.6–0.7 times as long as proximal mandibular width; lower face about [1.2] 1.0–1.4 times as broad as high, weakly convex centrally, polished, with fine sparse setiferous punctures; head in dorsal view with margin of gena flat behind the eyes, and its margin about [0.6] 0.4–0.6 times length of eye; ocelli moderately large, the lateral ones separated from compound eyes by [1.1] 1.0–1.3 times their own maximum diameter. ***Mesosoma*.** Pronotum without epomia; shelf-like projection, in dorsal view, more or less bilobed, subquadrangular, and in lateral view, slender with anterolateral corners weakly decurved; mesoscutum more or less slender, in dorsal view, smooth and polished, with notauli weakly impressed anteriorly; scutellum convex, not laterally carinate; mesopleuron highly polished, virtually impunctate; epicnemial carina reaching almost level of lower corner of pronotum; epicnemium with vestigial vertical carina near lower corner of pronotum; metapleuron convex, smooth and polished, with few sparse, fine bristles evenly spaced, without discernible submetapleural carina. Propodeum mediodorsally smooth and polished, with longitudinal carinae present only posteriorly and laterally with scattered fine bristles. Fore wing length [10.0] 10.0–11.0 mm; 1*cu-a* interstitial relative to *M&RS*; base of 1*m-cu&M* separated from *CU* by about length of 2*cu-a*; hind wing with distal abscissa of *CU* present and complete but weakly pigmented; first abscissa of *RS* subequal to *rs-m*. Tarsal claw with proximal lobe quadrangular, with claw apex slightly overtaking the distal margin of lobe. ***Metasoma*.** Tergite I about [1.4] 1.4–1.8 times as long as posteriorly broad, dorsally with lateromedian longitudinal carinae only discernible at extreme anterior part; sternite I with weak swelling near hind rim, and with weak median longitudinal ridge anteriorly; tergite II about [1.4] 1.4–1.7 times as long as posteriorly broad, highly polished, at most with only fine setiferous punctures laterally; tergite III about [1.3] 1.3–1.4 times as long as posteriorly broad, highly polished, at most with only fine setiferous punctures laterally; subgenital plate subquadrate. Ovipositor slightly slender, about [1.2] 1.1–1.3 times as long as hind tibia, posteriorly evenly tapered to sharp point.

##### Color.

Head black except 0.8 distal of clypeus yellowish; antennae brown; mouthparts pale, except apex of mandible brownish. Mesosoma orange, except posterior carinae of propodeum darkish brown. Metasoma orange, with posterior margins of tergites II–V narrowly black, tergites VI+ black. Fore leg orange, mid leg orange with tarsus brownish, hind leg darkish brown, with median region of tibia pale. Wings yellowish hyaline with apex weakly blackish, pterostigma yellow. Ovipositor darkish brown, with posterior and anterior parts pale.

##### Variation.

Some specimens present hind leg entirely darkish brown; metasoma orange with posterior margins of tergites II–V narrowly black only laterally and tergite VI orange with posterior margin black.

**Male.** Unknown.

##### Type material.

***Holotype*** ♀. Peru, CU [= Cusco], Cosñipata valley, Rocotal 13°07'00"S, 71°34'20"W, 2075 m., 23.X.2007, Malaise trap (C. Castillo leg.), MUSM. ***Paratypes***: idem holotype, but San Pedro, 13°03'22"S, 71°32'55"W, 1520 m., 1♀, ZMUT. Ecuador: R. Biol. San Francisco, 03°58'30"S, 79°04'25"W, 2000 m., 13.II–03.III.2009, Malaise trap (M. Pollet & A. Braekeleer leg.), EC/2009-36/MP&ADB-017 [code?], 2♀♀, RBINS.

##### Distribution.

Ecuador and Peru (Fig. 12).

##### Biological note.

Host unknown.

##### Etymology.

This species is named in honour of the Andean Inca empire.

##### Remarks.

*Polysphinctainca* sp. nov. closely resembles *A.sinearanea* Pádua, 2018 and *P.pichincha* sp. nov. mainly by color pattern, body orange with last metasomal tergites black. It differs from *P.sinearanea* by having epomia absent (present in *P.sinearanea*), and from *P.pichincha* sp. nov. by having malar space > 0.6 times as long as proximal mandibular width (malar space 0.4 times as long as proximal mandibular width in *P.pichincha* sp. nov.).

#### Polysphincta macroepomia

Taxon classificationAnimaliaHymenopteraIchneumonidae

Pádua & Sääksjärvi sp. nov.

F64991F5-104C-5F2B-839E-351AF1E1CE0B

http://zoobank.org/67A518F3-413E-4233-B77A-135CEDF17743

Fig. 5A–E

##### Diagnose.

*Polysphinctamacroepomia* sp. nov. can be distinguished from other species of the *P.dizardi* species-group by the combination of the following characters: (1) epomia present, 1.5 times length of proximal mandibular width (Fig. 5B); (2) malar space 0.6 times as long as proximal mandibular width (Fig. 5B); (3) fore wing vein 1*cu-a* interstitial relative to *M&RS* (Fig. 5A); (4) mesosoma orange, except metapleuron and propodeum darkish brown (Fig. 5A); (5) fore wing hyaline (Fig. 5A); (6) hind leg whitish with spot in proximal region of coxa, base of trochanter, longitudinal spot in subdistal region of inner and outer margin of femur, distal part of tibia and distal part of tarsus brownish (Fig. 5A); (7) metasoma darkish brown, with posterior margins of tergites II–V narrowly black (Fig. 5A, C); (8) ovipositor slightly slender, 1.7 times as long as hind tibia.

##### Description.

**Female.** Body about [7.0] mm. ***Head*.** Clypeus very weakly convex, posterior margin thin and flat centrally; malar space [0.6] times as long as proximal mandibular width; lower face about [1.1] times as broad as high, weakly convex centrally, polished, with fine sparse setiferous punctures; head in dorsal view with margin of gena very weakly convex behind the eyes, and its margin about [0.6] times length of eye; ocelli moderately large, the lateral ones separated from compound eyes by [1.0] times their own maximum diameter. ***Mesosoma*.** Pronotum with epomia distinct, about [1.5] times length of proximal mandibular width; shelf-like projection, in dorsal view, more or less straight, broader than long, and in lateral view, slender and slightly decurved in apex; mesoscutum robust, in dorsal view, smooth and polished, with notauli weakly impressed anteriorly; scutellum convex, not laterally carinate; mesopleuron highly polished, virtually impunctate; epicnemial carina reaching almost the level of lower corner of pronotum; epicnemium with vestigial vertical carina near lower corner of pronotum; metapleuron weakly convex, smooth and polished, with few sparse fine bristles, without discernible submetapleural carina. Propodeum mediodorsally smooth and polished, with longitudinal carinae present only posteriorly and with scattered fine bristles. Fore wing length [6.0] mm; 1*cu-a* interstitial relative to *M&RS*; base of 1*m-cu&M* separated from *CU* by about length of 2*cu-a*; hind wing with distal abscissa of *CU* present and complete but weakly pigmented; first abscissa of *RS* subequal to *rs-m*. Tarsal claw with proximal lobe quadrangular, with claw apex slightly overtaking distal margin of lobe. ***Metasoma*.** Tergite I about [1.4] times as long as posteriorly broad, dorsally with lateromedian longitudinal carinae only discernible at extreme anterior part; sternite I with weak swelling near hind rim, and with weak median longitudinal ridge anteriorly; tergite II [1.3] times as long as posteriorly broad, highly polished, at most with only fine setiferous punctures laterally; tergite III about [1.2] times as long as posteriorly broad, highly polished, at most with only fine setiferous punctures; subgenital plate subquadrate. Ovipositor slightly slender, [1.7] times as long as hind tibia, posteriorly evenly tapered to sharp point.

##### Color.

Head darkish brown except clypeus brownish; antennae brown; mouthparts white, except apex of mandible black. Mesosoma orange, except metapleuron and propodeum darkish brown. Metasoma entirely darkish brown, with posterior margins of tergites II–V narrowly black. Legs whitish, fore leg with femur, tibia and tarsus weakly yellowish; mid leg with femur and tibia and tarsus weakly yellowish, except final distal of tarsus brownish; hind leg with spot in proximal region of coxa, base of trochanter, longitudinal spot in subdistal region of inner and outer margin of femur, distal part of tibia and final distal of tarsus brownish. Wings hyaline, pterostigma brown. Ovipositor brown, with posterior portion whitish.

**Male.** Unknown.

##### Type material.

***Holotype*** ♀. Peru, CU [= Cusco], San Pedro, 1520 m., 13°03'22"S, 71°32'55"W, 22.IX.2007, Malaise trap 11 (C. Castillo leg.), MUSM.

##### Distribution.

Peru (Fig. 13).

##### Biological notes.

Host unknown.

##### Etymology.

The specific name refers to the long epomia, main characteristic of this species.

##### Remarks.

*Polysphinctamacroepomia* sp. nov. closely resembles *P.dizardi* Gauld, 1991 and *P.cosnipata* sp. nov. mainly by the coloration, mesosoma orange with metapleuron and propodeum blackish and metasoma brownish or blackish. However, it differs from both species by having epomia present (absent in *P.dizardi* and *P.cosnipata* sp. nov.).

#### Polysphincta organensis

Taxon classificationAnimaliaHymenopteraIchneumonidae

Pádua & Sääksjärvi sp. nov.

2A32E53E-1025-5A07-A18B-A8F49F135A44

http://zoobank.org/B43CD278-FD39-44F7-B453-5940E400CAA8

Fig. 6A–G

##### Diagnosis.

*Polysphinctaorganensis* sp. nov. can be distinguished from other species of the *P.dizardi* species-group by the combination of the following characters: (1) epomia absent (Fig. 6B); (2) malar space 0.4 times as long as proximal mandibular width (Fig. 6B); (3) fore wing vein 1*cu-a* interstitial relative to *M&RS* (Fig. 6A); (4) mesosoma orange with weak spot posteriorly in metapleuron and posterior carinae of propodeum brown (Fig. 6A); (5) wings hyaline (Fig. 6A); (6) hind leg whitish with inner region of coxa, trochanter proximally, trochantellus distally, femur proximally and distally, tibia proximally and distally, first tarsal segment distally, and remaining tarsal segments entirely blackish brown (Fig. 6A); (7) metasoma blackish with anterior margin centrally orange in tergite I, tergites II–VI with anterior and anterolateral margins whitish (Fig. 6A, C, G); (8) ovipositor slightly slender, 1.2–1.3 times as long as hind tibia.

##### Description.

**Female.** Body [8.5] 7.0–8.5 mm. ***Head*.** Clypeus weakly convex, posterior margin thin and straight centrally; malar space [0.4] times as long as proximal mandibular width; lower face about [0.9] 0.9–1.0 times as broad as high, weakly convex centrally, polished, with fine sparse setiferous punctures; head in dorsal view with margin of gena weakly convex behind the eyes, and its margin about [0.5] 0.45–0.5 times length of eye; ocelli moderately large, lateral ones separated from compound eyes by about [0.9] 0.9–1.0 times their own maximum diameter. ***Mesosoma*.** Pronotum without epomia; shelf-like projection, in dorsal view, more or less bilobed, subquadrangular, and in lateral view, slender and with anterolateral corners weakly decurved; mesoscutum robust, in dorsal view, smooth and polished, with notauli weakly impressed anteriorly; scutellum convex, not laterally carinate; mesopleuron highly polished, virtually impunctate; epicnemial carina reaching almost level of lower corner of pronotum; epicnemium with vestigial vertical carina near lower corner of pronotum; metapleuron convex, smooth and polished, with few sparse, fine bristles evenly spaced, without discernible submetapleural carina. Propodeum mediodorsally smooth and polished, with longitudinal carinae present only posteriorly and with scattered fine bristles. Fore wing length about [7.0] 5.0–7.0 mm; 1*cu-a* interstitial relative to *M&RS*; base of 1*m-cu&M* separated from *CU* by about length of 2*cu-a*; hind wing with distal abscissa of *CU* present and complete; first abscissa of *RS* subequal to *rs-m*. Tarsal claw with proximal lobe quadrangular, with claw apex slightly overtaking the distal margin of lobe. ***Metasoma*.** Tergite I about [1.5] times as long as posteriorly broad, dorsally with lateromedian longitudinal carinae only discernible at extreme anterior part; sternite I with weak swelling near hind rim, and with weak median longitudinal ridge anteriorly; tergite II about [1.2] 1.0–1.2 times as long as posteriorly broad, highly polished, at most with only fine setiferous punctures laterally; tergite III about [0.9] 0.9–1.1 times as long as posteriorly broad, highly polished, at most with only fine setiferous punctures; subgenital plate subquadrate. Ovipositor slender, about [1.3] 1.2–1.3 times as long as hind tibia, distally evenly tapered to sharp point.

##### Color.

Head black except 0.8 distal of clypeus yellowish; antennae brownish with scape and pedicel ventrally whitish; mouthparts whitish, except apex of mandible brownish. Mesosoma orange with weak spot posteriorly of metapleuron and posterior carinae of propodeum brown. Metasoma blackish with anterior margin centrally orange in tergite I, tergites II–VI with anterior and anterolateral margins whitish. Fore leg orange, mid leg orange with coxa, trochanter and trochantellus whitish and tarsus brownish, hind leg whitish with coxa inner region, trochanter proximal, trochantellus distal, femur proximally and distally, tibia proximally and distally, first tarsal segment distally, and remaining tarsal segments entirely blackish brown. Wings hyaline, pterostigma brown. Ovipositor darkish brown, with posterior and anterior part whitish.

**Male.** (Fig. 6G). Similar to female in structure and coloration, but body about 5.0 mm; malar space 0.3 times as long as proximal mandibular width; lower face about 1.15 times as broad as high; lateral ocelli separated from compound eyes by about 0.75 times their own maximum diameter; fore wing length about 4.0 mm.

##### Type materials.

***Holotype*** ♀. Brazil, RJ [= Rio de Janeiro], Teresópolis, PARNASO [= Parque Nacional Serra dos Órgãos], Pto. 6A, 868 m, 22°28'11.8"S, 43°00'05.3"W, I.2015, [Malaise trap] (R.F. Monteiro et al. leg.), DCBU. ***Paratypes***: idem holotype, but 1♀ and 1♂, MZUSP; idem, but 1♀ and 1♂, DCBU; idem, but 2♂♂, INPA; idem, but 1♂, INPA; idem but Pto. 8B, 1068 m, 22°27'09.0"S, 42°59'30.8"W, I.2015, 1♀, MZUSP; idem, but Pto. 9B, 1246 m, 22°26'55.1"S, 43°00'16.4"W, III.2015, 1♀, DCBU; idem, but Pto. 6B, 877 m, 22°28'11.5"S, 43°00'06.0"W, X.2015, 2♀♀, INPA; idem, but 1♀, MZUSP; idem, but Guapimirim, Pto. 4B, 540 m, 22°28'36.4"S, 42°59'30.7"W, XII.2014, 1♂, DCBU; idem, but Pto. 4A, 549 m, 22°28'36.5"S, 42°59'30.8"W, I.2015, 1♀, DCBU; idem, but 1♀, MZUSP.

##### Distribution.

Brazil (Fig. 11).

##### Biological note.

Host unknown.

##### Etymology.

The specific name refers to the type locality of this species Serra dos Órgãos, Rio de Janeiro state, Brazil.

##### Remarks.

*Polysphinctaorganensis* sp. nov. closely resembles *P.bonita* sp. nov. mainly by coloration: mesosoma entirely orange and propodeum brownish with some whitish in anterior part on tergites. It differs from *P.bonita* sp. nov. by having ovipositor >1.2 times as long as hind tibia and fore leg orange, mid leg orange with coxa, trochanter and trochantellus whitish and tarsus brownish, hind leg whitish with inner region of coxa, trochanter proximally, trochantellus distally, femur proximally and distally, tibia proximally and distally, first tarsal segment distally, and remaining tarsal segments entirely blackish brown (ovipositor 1.0 times as long as hind tibia and fore and mid leg orange, hind leg brownish, except coxa orange and a medium inner and outer region whitish in *P.bonita* sp. nov.).

#### Polysphincta pichincha

Taxon classificationAnimaliaHymenopteraIchneumonidae

Pádua, Sääksjärvi & Spasojevic sp. nov.

20A9114E-2566-5EAD-AA99-94C16FF6E0AF

http://zoobank.org/30D68683-592D-44E2-90EA-7E801DDAE203

Fig. 7A–E

##### Diagnosis.

*Polysphinctapichincha* sp. nov. can be distinguished from other species of the *P.dizardi* species-group by the combination of the following characters: (1) epomia absent (Fig. 7B); (2) malar space 0.4 times as long as proximal mandibular width (Fig. 7B); (3) fore wing vein 1*cu-a* more or less interstitial relative to *M&RS* (Fig. 7A); (4) mesosoma entirely orange (Fig. 7A); (5) wings yellowish hyaline (Fig. 7A); (6) hind leg orange with trochanter, apex distal and proximal of femur, tibia, except longitudinal spot pale in subdistal region of inner and outer margin and tarsus brownish (Fig. 7A); (7) metasoma orange, with posterior margins of tergites II–IV narrowly black, tergites V+ black (Fig. 7A, C); (8) ovipositor slender, 1.2 times as long as hind tibia.

##### Description.

**Female.** Body [13.5] mm. ***Head*.** Clypeus weakly convex, posterior margin thin and flat centrally; malar space [0.4] times as long as proximal mandibular width; lower face about [1.0] times as broad as high, weakly convex centrally, polished, with fine sparse setiferous punctures; head in dorsal view with margin of gena flat behind eyes, and its margin about [0.5] times length of eye; ocelli moderately large, lateral ones separated from compound eyes by [0.85] times their own maximum diameter. ***Mesosoma*.** Pronotum without epomia; shelf-like projection, in dorsal view, more or less bilobed, broader than long, and, in lateral view, slender and with anterolateral corners weakly decurved; mesoscutum slender, in dorsal view, smooth and polished, with notauli weakly impressed anteriorly; scutellum convex, not laterally carinate; mesopleuron highly polished, virtually impunctate; epicnemial carina reaching almost level of lower corner of pronotum; epicnemium with vestigial vertical carina near lower corner of pronotum; metapleuron convex, smooth and polished, with few sparse, fine bristles evenly spaced, without discernible submetapleural carina. Propodeum mediodorsally smooth and polished, with longitudinal carinae present only posteriorly and laterally with scattered fine bristles. Fore wing length [10.0] mm; 1*cu-a* more or less interstitial relative to *M&RS*; base of 1*m-cu&M* separated from *CU* by more than length of 2*cu-a*; hind wing with distal abscissa of *CU* present and complete; first abscissa of *RS* subequal to *rs-m*. Tarsal claw with proximal lobe quadrangular, with claw apex slightly overtaking the distal margin of lobe. ***Metasoma*.** Tergite I about [1.8] times as long as posteriorly broad, dorsally with lateromedian longitudinal carinae only discernible at extreme anterior part; sternite I with weak swelling near hind rim, and with weak median longitudinal ridge anteriorly; tergite II about [1.6] times as long as posteriorly broad, highly polished, at most with only fine setiferous punctures laterally; tergite III [1.4] times as long as posteriorly broad, highly polished, at most with only fine setiferous punctures laterally; subgenital plate subquadrate. Ovipositor slightly slender, about [1.2] times as long as hind tibia [without apex].

##### Color.

Head black, except posterior margin of clypeus whitish; antennae brown; mouthparts pale, except apex of mandible brownish. Mesosoma orange. Metasoma orange, with posterior margins of tergites II–IV narrowly black, tergites V+ black. Legs orange, the mid leg with tarsus brownish, hind leg with trochanter, apex distal and proximal of femur, tibia, except longitudinal spot pale in subdistal region of inner and outer margin and tarsus brownish. Wings yellowish hyaline, pterostigma yellow.

**Male.** Unknown.

##### Type material.

***Holotype*** ♀. Ecuador, Pichincha, Nambillo Valley near Mindo, 1450 m, 26.VI.1987 (M. Cooper leg.), #2005-152, BMNH.

##### Distribution.

Ecuador (Fig. 11).

##### Biological note.

Host unknown.

##### Etymology.

The specific name (in apposition) refers to type locality of this species, Pichincha province, Ecuador.

##### Remarks.

*Polysphinctapichincha* sp. nov. closely resembles *A.sinearanea* Pádua, 2018 and *P.inca* sp. nov. mainly by coloration with body orange and the last tergites black. It differs from *P.sinearanea* by having epomia absent (present in *P.sinearanea*), and from *P.inca* sp. nov. by having malar space 0.4 times as long as proximal mandibular width (malar space >0.6 times as long as proximal mandibular width in *P.inca* sp. nov.).

#### Polysphincta shabui

Taxon classificationAnimaliaHymenopteraIchneumonidae

Gauld, 1991

CA80AE9E-A002-5252-A7CB-2D57690611BB

Fig. 8A–F

Polysphincta shabui Gauld, 1991: 314. Holotype ♀, Costa Rica (MNCR).

##### Diagnosis.

*Polysphinctashabui* can be distinguished from other species of the *P.dizardi* species-group by the combination of the following characteristics: (1) epomia absent (Fig. 8B); (2) malar space 0.6 times as long as proximal mandibular width (Fig. 8B); (3) fore wing vein 1*cu-a* more or less interstitial relative to *M&RS* (Fig. 8A); (4) mesosoma entirely reddish brown (Fig. 8A); (5) wings hyaline (Fig. 8A); (6) hind leg orange with femur, tibia and tarsus darkish brown (or femur reddish orange) (Fig. 8A); (7) metasoma entirely darkish brown or darkish brown with tergites I–III reddish orange with posterior margins narrowly black (Fig. 8A, C); (8) ovipositor slender, 1.2–1.4 times as long as hind tibia.

##### Comments.

Additional characters to the original description (♀) are as follows: body about [10.5–11.0] 10.5–14.0; head in dorsal view with margin of the gena flat behind the eyes, and its margin [0.5] 0.5–0.6 times length of eye; shelf-like projection, in dorsal view, developed anterolaterally in apex, the apex bilobed, and in lateral view, with anterolateral part in apex rounded and weakly decurved; mesoscutum robust, in dorsal view; tarsal claw with proximal lobe quadrangular, with claw apex slightly overtaking the distal margin of lobe.

##### Distribution.

Costa Rica and Brazil (Fig. 12).

##### Biological notes.

Host unknown.

##### Materials examined.

***Paratypes*:** Costa Rica, Limón Pv., 16 km, W. Guápiles, 400 m, V.1989 (without collector), 1♀, BMNH; idem, but Heredia Pv., Braulio Carrillo, 9.5 km, E. of El Tunel, 1000 m, X–XI.1989, 1♀, BMNH. **Costa Rica**: Cartago Pv., Cachí, 1200 m, II.1996 (Chaves leg.), 1♀, BMNH; Ptas Pv., San Vito, Las Alturas, 1500 m, V.1992 (K. Gaston leg.), 1♀, BMNH.

#### Polysphincta sinearanea

Taxon classificationAnimaliaHymenopteraIchneumonidae

Pádua, 2018

750DFA77-C7F5-5D72-8023-105CE10B4DAC

Fig. 9A–E

Polysphincta sinearanea Pádua, 2018 *in*Kloss et al. 2018: 102. Holotype ♀, Brazil (INPA).

##### Diagnosis.

**Figure 9.**
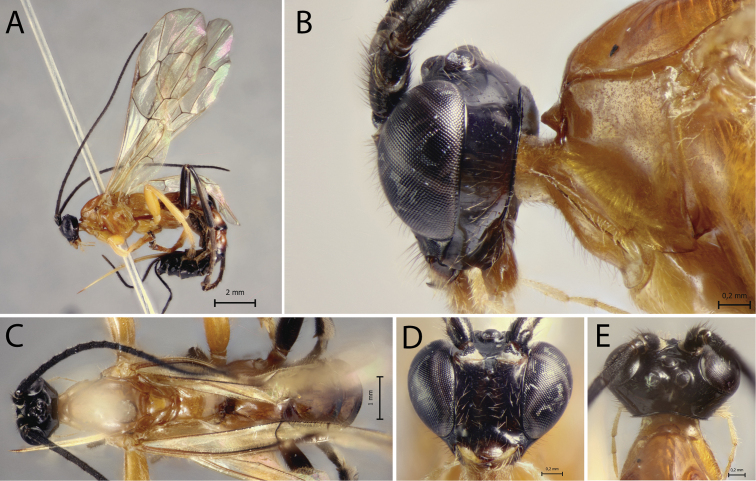
*Polysphinctasinearanea* Pádua, 2018, ♀, paratype **A** habitus, lateral view **B** head and pronotum, lateral view **C** habitus, dorsal view **D** face, anterior view **E** head and shelf-like projection, dorsal view.

*Polysphinctasinearanea* can be distinguished from other species of the *P.dizardi* species-group by the combination of the following characters: (1) epomia present, about 0.9 times length of proximal mandibular width (Fig. 9B); (2) malar space 0.5–0.6 times as long as proximal mandibular width (Fig. 9B); (3) fore wing vein 1*cu-a* more or less interstitial relative to *M&RS* (Fig. 9A); (4) mesosoma entirely orange (Fig. 9A); (5) fore wing very slightly yellowish hyaline (Fig. 9A); (6) hind leg orange with femur, tibia and tarsus brownish (Fig. 9A); (7) metasoma orange, with posterior margins of tergites II–IV narrowly black, posterior half of tergite V black, and tergites VI+ black (Fig. 9A, C); (8) ovipositor robust, 1.5 times as long as hind tibia.

##### Comments.

Additional characters to the original description (♀) are as follows: head in dorsal view with margin of the gena flat behind the eyes, and its margin [0.7] times length of eye; shelf-like projection, in dorsal view, weakly developed in the anterolateral part of apex, the apex more or less straight, not bilobed, and in lateral view, with anterolateral part in apex more or less rounded and not decurved; mesoscutum robust, in dorsal view; tarsal claw with proximal lobe quadrangular, with claw apex slightly overtaking the distal margin of lobe; ovipositor robust.

##### Distribution.

Brazil (Fig. 12).

##### Biological notes.

Parasitoid of the spider species *Metazygialaticeps* (O. Pickard-Cambridge, 1889) (Kloss et al. 2018).

##### Materials examined.

***Holotype*:** Brazil, Espírito Santo, Cariacica, Res. [= Reserva] Biológica de Duas Bocas, 26.I.2017, parasitizing *M.laticeps* (T.G. Kloss leg.), INPA. ***Paratypes***: same data of holotype, 1♀ and 2♂♂ (one with the last metasomal segments extracted), INPA; Minas Gerais, Viçosa, Mata do Prof. Chaves (Silvestre), V.2017, parasitizing *M.laticeps* (T.G. Kloss leg.), 1♀ and 1♂, INPA.

#### Polysphincta teresa

Taxon classificationAnimaliaHymenopteraIchneumonidae

Pádua & Sääksjärvi sp. nov.

E2EA541F-EEC3-5FDC-8C18-84C184EB0268

http://zoobank.org/E22AD4A9-CB81-467A-83F5-A0BB902659B6

Fig. 10A–F

##### Diagnose.

**Figure 10.**
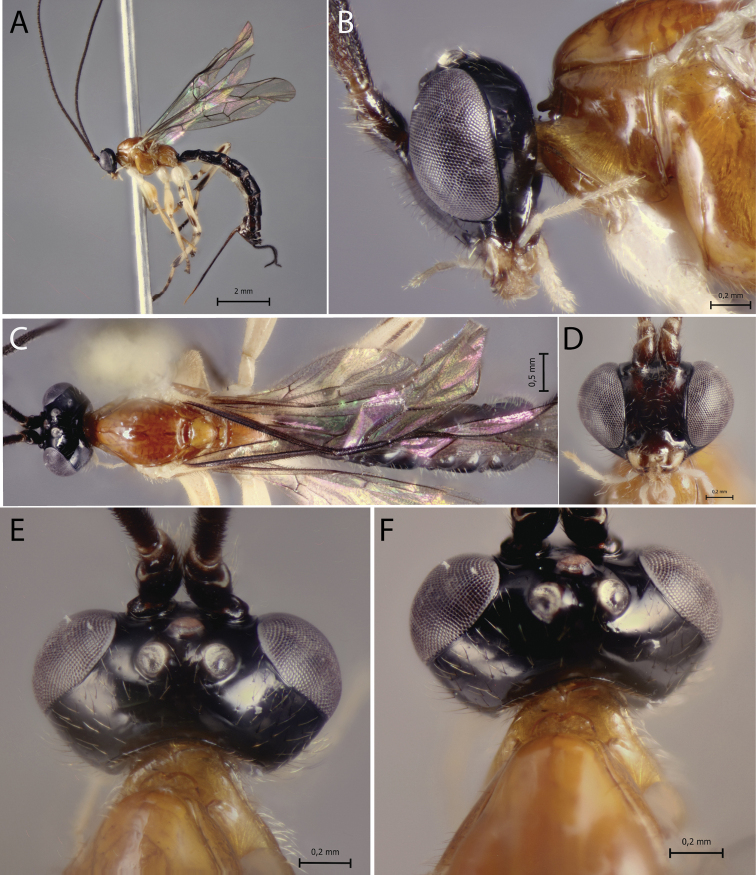
*Polysphinctateresa* sp. nov., ♀, holotype **A** habitus, lateral view **B** head and pronotum, lateral view **C** habitus, dorsal view **D** face, anterior view **E** head, dorsal view **F** shelf-like projection, dorsal view.

*Polysphinctateresa* sp. nov. can be distinguished from other species of the *P.dizardi* species-group by the combination of the following characters: (1) epomia present, about 0.9–1.0 times length of proximal mandibular width (Fig. 10B); (2) malar space 0.4–0.6 times as long as proximal mandibular width (Fig. 10B); (3) fore wing with vein 1*cu-a* postfurcal relative to *M&RS* (0.25–0.35 times its own length) or 1*cu-a* more or less interstitial relative to *M&RS* (Fig. 10A); (4) mesosoma orange, except posterior carinae of propodeum darkish brown (Fig. 10A); (5) fore wing hyaline (Fig. 10A); (6) hind leg whitish with proximal region of trochanter, longitudinal spot in subdistal region of inner and outer margin of femur, distal part of tibia and first tarsal segment distally and remaining tarsal segments brownish (Fig. 10A); (7) metasoma darkish brown, with posterior margins of tergites II–V narrowly black (Fig. 10A, C); (8) ovipositor slightly slender, [1.7] 1.4–1.7 times as long as hind tibia.

##### Description.

**Female.** Body [7.75] 7.0–9.0 mm. ***Head*.** Clypeus weakly convex, posterior margin thin and straight centrally; malar space [0.4] 0.4–0.6 times as long as proximal mandibular width; lower face about [1.1] 0.9–1.1 times as broad as high, weakly convex centrally, polished, with fine sparse setiferous punctures; head in dorsal view with margin of gena very weakly convex behind eyes, and its margin [0.55] 0.4–0.6 times length of eye; ocelli moderately large, lateral ones separated from compound eyes by [0.9] 0.75–1.0 times their own maximum diameter. ***Mesosoma*.** Pronotum with epomia distinct, about [0.9] 0.9–1.3 times length of proximal mandibular width; shelf-like projection, in dorsal view, developed in anterolateral part of apex, apex bilobed, and in lateral view, with anterolateral part in apex rounded and weakly decurved; mesoscutum robust, in dorsal view, smooth and polished, with notauli weakly impressed anteriorly; scutellum convex, not laterally carinate; mesopleuron highly polished, virtually impunctate; epicnemial carina reaching almost level of lower corner of pronotum; epicnemium with vestigial vertical carina near lower corner of pronotum; metapleuron convex, smooth and polished, with sparse, fine bristles evenly spaced, without discernible submetapleural carina. Propodeum mediodorsally smooth and polished, with longitudinal carinae present only posteriorly and laterally with scattered fine bristles. Fore wing length [6.0] 5.0–7.0 mm; 1*cu-a* postfurcal relative to *M&RS* by [0.35] 0.25–0.35 times its own length; base of 1*m-cu&M* separated from *CU* by more than length of 2*cu-a*; hind wing with distal abscissa of *CU* present and complete but weakly pigmented; first abscissa of *RS* subequal to *rs-m*. Tarsal claw with proximal lobe quadrangular, with claw apex slightly overtaking distal margin of lobe. ***Metasoma*.** Tergite I [1.1] 1.1–1.7 times as long as posteriorly broad, dorsally with lateromedian longitudinal carinae only discernible at extreme anterior part; sternite I with weak swelling near hind rim, and with weak median longitudinal ridge anteriorly; tergite II about [1.3] 1.1–1.3 times as long as posteriorly broad, highly polished, at most with only fine setiferous punctures laterally; tergite III [1.0] 1.0–1.3 times as long as posteriorly broad, highly polished, at most with only fine setiferous punctures laterally; subgenital plate subquadrate. Ovipositor slightly slender, [1.7] 1.4–1.7 times as long as hind tibia, posteriorly evenly tapered to sharp point.

##### Color.

Head black except lower face and clypeus brownish; antennae brown, except apex of scape and pedicel whitish; mouthparts white, except apex of mandible black. Mesosoma orange, except posterior carinae of propodeum darkish brown. Metasoma entirely darkish brown, with posterior margins of tergites II–V narrowly black. Legs whitish, fore leg with 0.7 distal of femur, tibia and tarsus weakly rufescent; mid leg with 0.3 distal of femur and tibia weakly rufescent, 0.2 distal of tarsomere I, distal half of tarsomere II, 0.8 distal tarsomere III and tarsomeres IV+ brownish; hind leg with proximal region of trochanter, longitudinal spot in subdistal region of inner and outer margin of femur, distal part of tibia and first tarsal segment distally and remaining tarsal segments brownish. Wings hyaline, pterostigma brown. Ovipositor brown, with anterior and posterior portions slightly whitish.

**Male.** Unknown.

##### Variation.

Some specimens with clypeus whitish and fore and mid legs with femur and tibia whitish, others have the fore leg entirely orange; the mid leg orange with tarsomeres brownish; the hind leg whitish, with inner margin of coxa, trochanter, trochantellus, proximal region and longitudinal spot in subdistal region of inner and outer margin of femur, proximal and distal part of tibia and all tarsus darkish brown.

##### Type materials.

***Holotype*** ♀. Brazil, RJ [= Rio de Janeiro], Teresópolis, PARNASO [= Parque Nacional Serra dos Órgãos], Pto. 9A, 1236 m, 22°26'57.8"S, 43°00'13.7"W, I.2015, [Malaise trap] (R.F. Monteiro et al. leg.), DCBU. ***Paratypes***: same data of holotype, 2♀♀, DCBU; idem, but Pto. 11A, 1681 m, 22°27'07.9"S, 43°00'53.8"W, I.2015, 2♀♀, MZUSP; Pto. 11B, 1649 m, 22°27'03.7"S, 43°00'54.0"W, I.2015, 1♀, DCBU; idem, but Pto. 7A, 952 m, 22°27'24.8"S, 42°59'07.2"W, IX.2015, 1♀, MZUSP; idem, but Pto. 10A, 1444 m, 22°26'51.0"S, 43°00'46.4"W, XI.2015, 1♀, INPA; idem, but Pto. 12A, 1812 m, 22°27'18.2"S, 43°00'58.9"W, 1♀, INPA; idem, but Guapimirim, Pto. 3A, 332 m, 22°29'40.5"S, 42°59'52.6"W, 1♀, DCBU; SP [= São Paulo], Luiz Antônio, Est. Ecológica de Jataí, Mata ciliar, Point 1, 21°36'47.00"S, 47°49'49.04"W, 30.I.2008, Light trap (Lara and team leg.), INPA; Amazonas, Manaus, WWF, Reserve 1208, Rede Central Norte, 12.XII.1984, Malaise trap (Bert Klein leg.) 1♀, INPA; MG [= Minas Gerais], Belo Horizonte, Estação Ecológica, 19°52'30"S, 43°58'20"W, 842 m, 02.VI.1999 (A.F. Kumagai leg.), 1♀, IHY 1500544, UFMG; idem, but Capitólio, Trilha do Sol, Ponto III, 01.VI.2012, Malaise trap (J.F. Nunes and team leg.), 1♀, INPA; [Santa Catarina], Nova Teutônia, 27°11'S, 52°23'W, 30.VIII.1938 (Fritz Plaumann leg.), 1♀ [without hind legs], BMNH.

##### Distribution.

Brazil (Fig. 13).

##### Biological note.

Host unknown.

##### Etymology.

The specific name (in apposition) refers to the “Cidade de Teresa”, informal name of the type locality, Teresópolis, Rio de Janeiro state, Brazil.

##### Remarks.

*Polysphinctateresa* sp. nov. closely resembles *P.shabui* Gauld, 1991 mainly by coloration, with mesosoma entirely orange and propodeum blackish with anterior parts whitish. It differs from *P.shabui* by having epomia present (absent in *P.shabui*).

**Figure 11.**
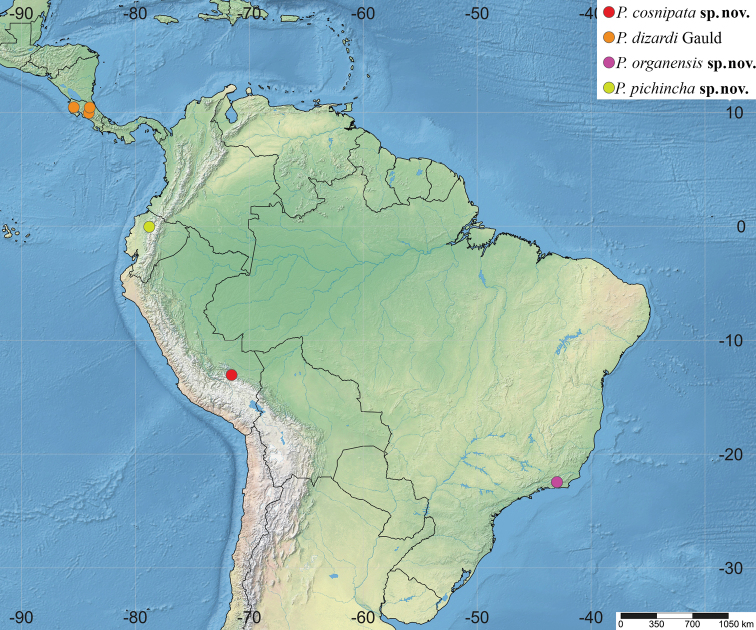
Distribution of *Polysphinctadizardi* group in the Neotropical Region.

**Figure 12.**
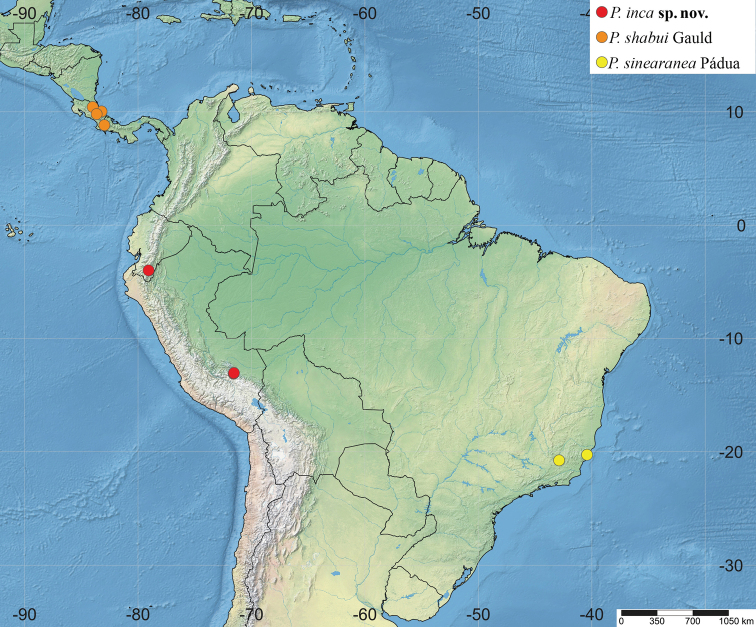
Distribution of *Polysphinctadizardi* group in the Neotropical Region.

**Figure 13.**
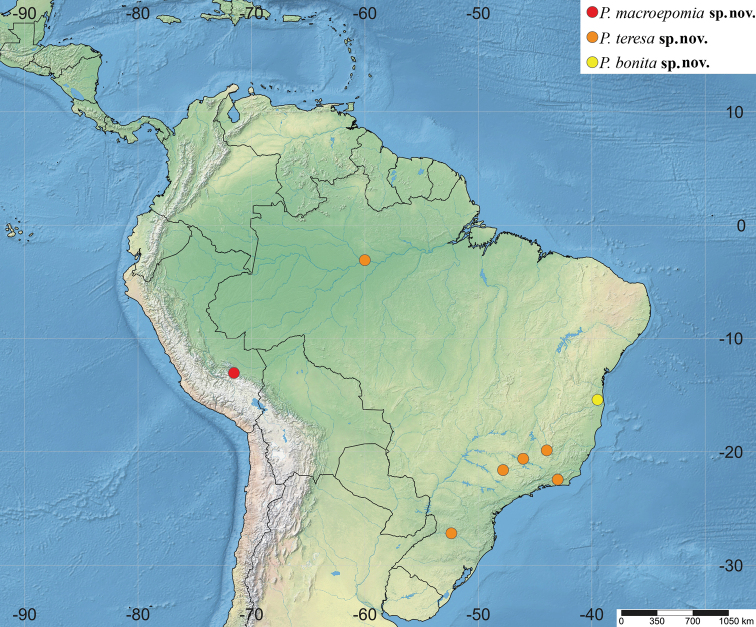
Distribution of *Polysphinctadizardi* group in the Neotropical Region.

## Discussion

Gauld (1991) characterized the *P.dizardi* species-group mainly by the shelf-like projection present mediodorsally on the pronotum. He also observed that the epomia was absent in most of the species (but present in one undescribed Brazilian species).

Pádua in Kloss et al. (2018) recently described a new species from Brazil (*P.sinearanea*) which is characterized by a strong epomia (Fig. 5B). In the present work, we described two additional new species which both have the epomia present (*P.macroepomia* sp. nov. and *P.teresa* sp. nov.). Thus, the *P.dizardi* species-group may no longer be defined by the absence of epomia.

We also studied the shape of the pronotal shelf and noted that it may be used in separating the species from each other. The shelf-like structure of the pronotum in *P.dizardi* species-group is a strong projection in the anterolateral part of the pronotal apex. We have also studied some undescribed species of *Polysphincta* from southeastern Brazil that possess a strong prominence in the same region of the pronotum. However, this structure is not developed into a strong shelf-like projection in those species. Therefore, we have not included those species in the present work, but we will describe them in a separate study in the future.

Gauld and Dubois (2006) proposed that the *P.dizardi* species-group could be a new polysphinctine genus that could be described when the tropical diversity of the group becomes better known. Given the non-declining rates of discovery and description of new polysphinctine species in the Neotropics (Kloss et al. 2018; Pádua et al. 2019, 2020a, b; Sobczak et al. 2019), we refrain at present from splitting the genus *Polysphincta*. We will firstly continue filling in the gap in biodiversity knowledge of *Polysphincta* of South America.

## Supplementary Material

XML Treatment for Polysphincta bonita

XML Treatment for Polysphincta cosnipata

XML Treatment for Polysphincta dizardi

XML Treatment for Polysphincta inca

XML Treatment for Polysphincta macroepomia

XML Treatment for Polysphincta organensis

XML Treatment for Polysphincta pichincha

XML Treatment for Polysphincta shabui

XML Treatment for Polysphincta sinearanea

XML Treatment for Polysphincta teresa
